# Characterization of photocatalytic TiO_2_ powder under varied environments using near ambient pressure X-ray photoelectron spectroscopy

**DOI:** 10.1038/srep43298

**Published:** 2017-02-27

**Authors:** Padmaja Krishnan, Minghui Liu, Pierre A. Itty, Zhi Liu, Vanessa Rheinheimer, Min-Hong Zhang, Paulo J. M. Monteiro, Liya E. Yu

**Affiliations:** 1 Department of Civil and Environmental Engineering, National University of Singapore, Singapore 119260, Singapore; 2 Department of Civil and Environmental Engineering, University of California, Berkeley, California 94720, USA; 3 Advanced Light Source, Lawrence Berkeley National Laboratory, Berkeley, California 94720, USA

## Abstract

Consecutive eight study phases under the successive presence and absence of UV irradiation, water vapor, and oxygen were conducted to characterize surface changes in the photocatalytic TiO_2_ powder using near-ambient-pressure X-ray photoelectron spectroscopy (XPS). Both Ti 2p and O 1s spectra show hysteresis through the experimental course. Under all the study environments, the bridging hydroxyl (OH_br_) and terminal hydroxyl (OH_t_) are identified at 1.1–1.3 eV and 2.1–2.3 eV above lattice oxygen, respectively. This enables novel and complementary approach to characterize reactivity of TiO_2_ powder. The dynamic behavior of surface-bound water molecules under each study environment is identified, while maintaining a constant distance of 1.3 eV from the position of water vapor. In the dark, the continual supply of both water vapor and oxygen is the key factor retaining the activated state of the TiO_2_ powder for a time period. Two new surface peaks at 1.7–1.8 and 4.0–4.2 eV above lattice oxygen are designated as peroxides (OOH/H_2_O_2_) and H_2_O_2_ dissolved in water, respectively. The persistent peroxides on the powder further explain previously observed prolonged oxidation capability of TiO_2_ powder without light irradiation.

Titanium dioxide (TiO_2_) is one of the most commonly used photocatalysts with wide modifications for numerous applications. To date, abundant experimental studies have reported atomic-level observations of TiO_2_ surfaces as thin films or single crystals using various tools, such as scanning tunneling microscopy (STM) and electron paramagnetic resonance (EPR). Most findings were summarized by Hendersen, with the details given in the references therein^1^,^2^,^3^. These articles have successfully advanced insights into the changes in the lattice and surface mechanisms of specific types of TiO_2_ crystal faces; yet, more understandings of the surface characteristics of commercially available TiO_2_ powder are needed to elucidate unique observations of actual applications under the ambient environment, such as disinfection by photocatalytic TiO_2_ powder in the dark.

Non-intrusive direct monitoring of activation and changes in bulk TiO_2_ powder under a more realistic environment are needed to investigate key issues, such as how the activated TiO_2_ can most effectively mineralize airborne pollutants. Currently, the effects of airborne components, such as moisture, on TiO_2_ performance are examined indirectly based on observations of the decrease in reactants or mineralization products (e.g., CO_2_). While X-ray photoelectron spectroscopy (XPS) has been a commonly employed tool to study surface properties, the high vacuum environment deters the possibility of examining semi-volatile and volatile species on surface, giving a less than complete picture. Superior to the conventional ultra-high vacuum XPS, the near-ambient pressure XPS coupled with the synchrotron light source provides a unique capability of *in situ* investigating surface characteristics (e.g. surface-adsorbed gases) with much higher sensitivity and resolution. It also enables one to study surface-environment interactions in real time by introducing desired components, such as gases, to obtain new findings and insights about surface changes. Although it is an ideal tool for direct observation of the sample surface, few articles have studied selected TiO_2_ single crystals with little effort made to examine the bulk photocatalytic TiO_2_ powder under conditions involving light irradiation, oxygen, and water vapor.

To address these needs, we employed an advanced XPS with synchrotron radiation light source to investigate the surface changes of bulk photocatalytic TiO_2_ powder under near ambient pressure with the successive supply and termination of UV irradiation, water moisture and oxygen through eight sequential experimental phases. The Ti 2p, O 1s and C 1s spectra were examined to show how changes in the environment affect the surface properties of photocatalytic TiO_2_ powder. This unique experiment design allowed us to obtain various results that are reported for the first time. Below, we first provide the general trends in the core Ti 2p and O 1s spectra before detailed discussion of major findings in individual study phases, including differentiating surface hydroxyl groups, delineating dynamics of physisorbed water, and elucidating continual redox capability of TiO_2_ powder in the dark.

## Results and Discussion

**Figure 1:**
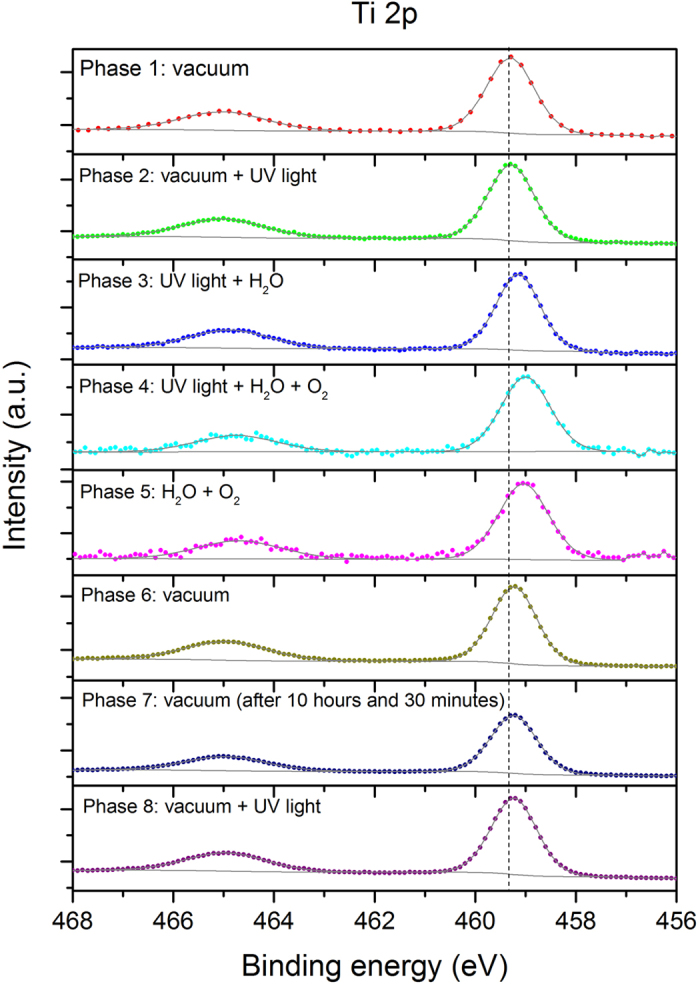
Hysteresis trend in Ti 2p spectra through eight study phases. The dashed vertical line is added to guide the visual observation.

**Figure 2:**
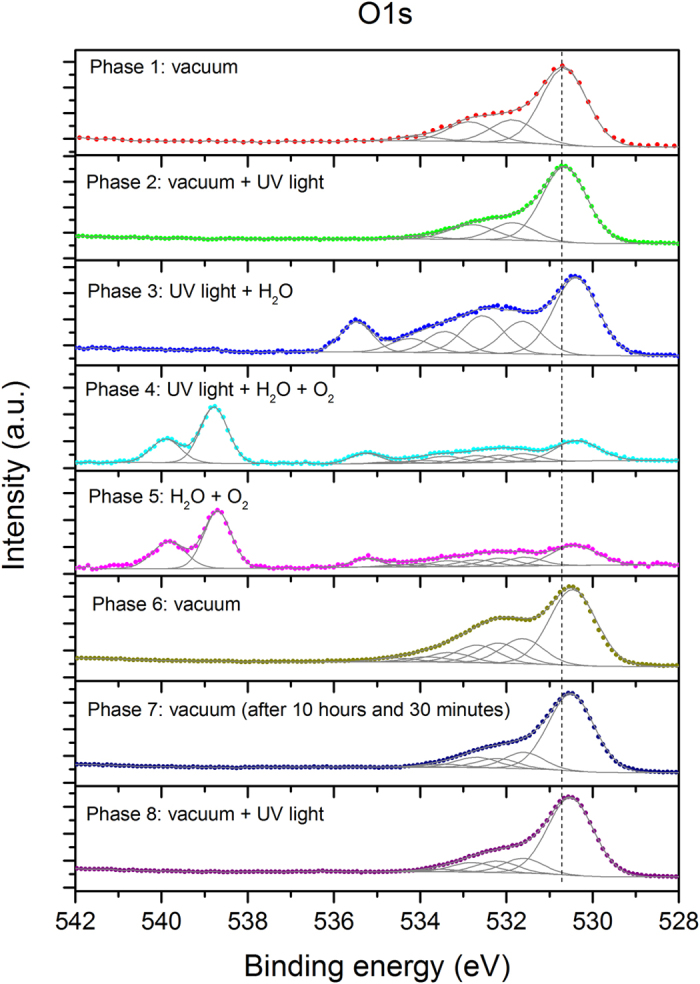
Hysteresis trend in O 1s spectra through eight study phases. The dashed vertical line is added to guide the visual observation.

The core peaks of the Ti 2p and O 1s XPS spectra exhibited hysteresis trends through the individual eight phases (Figs 1 and 2), which is observed for the first time via *in situ* XPS under varied ambient environment. In general, the peaks shifted to lower BE following the sequential supply of UV irradiation, water vapor, and oxygen, reaching the lowest value during phase 4 and phase 5. The decreasing trend was reversed upon termination of the supply of both gases in the dark during phase 6, arriving at the final position in phases 6–8, which is similar to the initial condition in phase 1 (Figs 1 and 2).

New features in the O 1s spectra are observed along with the hysteresis trend and are more responsive to the change in the ambient environment than the Ti 2p spectra. The accumulated decrease in the Ti 2p core peaks from phase 1 to phase 4 is significant, although the Ti 2p core peaks show a marginal decrease in the BE between every two adjacent phases with water vapor and oxygen supplied successively (Fig. 1). The notable accumulated change in the Ti 2p core peaks during the first four phases is confirmed by the pivotal shift in phase 6, when the termination of water vapor and oxygen supply in the dark reversed the trend and increased the BE of the core peaks by ~0.2 eV (Fig. 2). This is the largest incremental change in a single phase for the Ti 2p core peaks, which offsets all the previous progressive decreases in the BE positions, returning the Ti 2p core peaks to their original positions (phase 6, Fig. 2). Unlike the Ti 2p spectra, at the end of the experiment, the O 1s spectra exhibited persistent additions (Fig. 2), reflecting the lasting impacts of the experimental cycle on the powder surface. A more detailed discussion is given below regarding the changes in both the Ti 2p and O 1s spectra coupled with the C 1s spectra for individual study phases.

### TiO_2_ under vacuum in a dark environment (Phase 1)

Under a vacuum and dark environment, the two identified Ti 2p peaks at 459.3 (Ti 2p_3/2_) and 465.0 eV (Ti 2p_1/2_) (Fig. 2) are similar to the published locations at 458.6–459.5 eV and 463.0–464.8 eV, respectively^4^,^5^,^6^,^7^,^8^,^9^. The two major O 1s peaks are positioned below 535.0 eV (Fig. 2), the region for surface species^10^. The hump shape of the O 1s spectrum indicates the presence of imbedded surface species, which were resolved to four peaks at 530.7, 531.9, 532.9, and 534.0 eV through peak deconvolution (Fig. 2). The first peak at 530.7 eV represents lattice O bound to Ti^4+^, agreeing with the previously published results ranging from 529.7–530.7 eV^4^,^5^,^7^,^8^,^9^,^11^,^12^,^13^,^14^. Three other peaks result from the influence of water molecules on the sample surface, in the order of the OH group with oxygen at the bridging oxygen site (OH_br_, 531.9 eV), the OH group as a terminal group (OH_t_, 532.9 eV) with oxygen attached to the five-coordinated Ti^4+^ with an O-Ti^4+^ covalent bond, and water molecules on the powder surface (534.0 eV) (Fig. 2).

When the study chamber was dry during phase 1, water molecules could inherently adsorb on samples approximately 3.3 eV higher than lattice oxygen (Table 1), agreeing with the published data reporting a difference of 2.0–3.8 eV between surface-adsorbed water and lattice oxygen^5^,^14^,^15^,^16^. The BE position of OH_br_ and OH_t_ on the P25 powder was differentiated at 1.2 eV and 2.2 eV above lattice oxygen in this study. While most relevant XPS studies attribute 531.5–532.4 eV to hydroxyl groups on the sample surface without distinguishing OH_br_ from OH_t_^4^,^5^,^7^,^12^,^17^,^18^, the given BE position of the unspecified OH groups is generally 1.1–2.8 eV higher than lattice oxygen. This supports our present study showing the un-deconvoluted OH groups (OH_br_ and OH_t_ together) at 1.4–1.8 eV above lattice oxygen. In addition, our deconvoluted OH_br_ positioning approximately 1.2 eV higher than lattice oxygen (Table 1), corresponding to 531.9 eV (Fig. 2), is consistent with Ketteler *et al*.^5^ who suggested that the OH_br_ peak on rutile (110) was positioned 1.1–1.6 eV higher than the lattice O 1s peak. Thus, isolating the speciated OH_br_ peak from the grouped position of hydroxyls enabled us to identify the OH_t_ peak at 2.2 eV above lattice oxygen (Table 1) or at 532.9 eV on the P25 powder in this phase (Fig. 2).

The identified OH_br_ and OH_t_ peaks during phase 1 encompass organic compounds because inherent organic contaminants on the sample surface are inevitable^19^, similar to surface-adsorbed water molecules. The presence of organic contaminants on our powder sample was examined based on the C 1s spectra with non-oxygenated C (C-C and C-H) at 284.8 eV. Relative to this peak, we identified carbonyl C (C=C), ester carbon (C-O) and carboxylate carbon (C=O) at BE 0.8 eV lower, 0.9 eV higher, and 1.9 eV higher, respectively (Supplementary Figure S1(a)), consistent with the positions reported in the literature^14^,^20^,^21^. The corresponding O 1s position of organics on samples exposed to air were located between 531.8 and 533.0 eV^5^,^20^,^21^,^22^, which could imbed under the overall hydroxyl peak, ranging from 529.9–534.8 eV in our study, affecting the deconvoluted positions of the hydroxyl peaks during phase 1. This interference on the deconvoluted position of the hydroxyl groups is further examined when the organic species diminished in the later phases, as discussed in the last section.

### Photocatalytic activation of TiO_2_ (Phase 2)

When UV irradiation was supplied (phase 2), little change in the Ti 2p core peaks was observed (Fig. 1), consistent with the study of Simonsen *et al*.^7^, who reported negligible effects of UV light on TiO_2_ thin films. Similar to the Ti 2p spectrum in this phase, there are no observable changes in the individual O 1s peaks (Fig. 2) although some surface carbonaceous contaminants were decomposed because the C 1s spectrum during phase 2 decreased in 282.5–287.5 eV (Supplementary Figure S1(b)), indicating a decrease in the carbonaceous functional groups, such as C=C, C-H, C-O, and C=O, which can undergo photolysis^21^.

### Effects of water vapor (Phase 3)

The addition of 0.2 Torr water vapor under UV irradiation lowered the core peaks of Ti 2p (phase 3, Fig. 1) halfway, reaching the lowest peak position during the entire experimental course. This differs from the negligible changes on R(110) film reported by Ketteler *et al*.^5^ who administered only 0.1 mTorr water vapor without UV irradiation, which could be too little to cause observable effects. Being more responsive to changes in the environment than Ti 2p, the O 1s spectrum shifted substantially, and two new peaks emerged at 533.4 and 535.5 eV (phase 3, Fig. 2). The first three O 1s peaks of lattice oxygen, OH_br_ and OH_t_ were lowered by more than 0.2 eV from their positions in phase 2 (Fig. 2) because the supplied water vapor increased the pressure in the study chamber and enhanced the amount of water molecules adsorbed on the powder. The supplied water vapor is expected to increase the entropy and decrease the Gibbs free energy in the system due to the “pressure factor” specified by Salmeron *et al*.^23^. The pressure factor exerted by the 0.2 Torr water vapor in our study is thus estimated to lower the BE by approximately 0.2 eV, consistent with the shift of the first three O 1s peaks in this phase.

In contrast to the lowered BE of the first three O 1s peaks, the surface-adsorbed water molecules increased by 0.2 eV reaching 534.2 eV (Fig. 2). Although the XPS peak of water adsorbed on the R(110) surface lowered its BE with increasing water coverage^5^, the amount of water vapor (1 Torr at 270 K) supplied in the study was more than five times the amount in our current work. On the other hand, changing the amount of water vapor from 0.45 to 4.5 Torr in another study did not cause notable changes in the water molecules adsorbed on stoichiometric A(101)^24^. These results demonstrate that the behavior of surface-adsorbed water molecules on different crystal planes varies, and can at times depend on the amount of water vapor supplied. Hence, the observation of single-crystal planes under a controlled environment may be inapplicable to the *in situ* properties of water molecules on P25 TiO_2_ powder, which comprises many crystal facets as “platforms” for diverse changes in surface-adsorbed water under altered ambient conditions.

In phase 3, the surface-adsorbed water molecules reached the highest BE position, lowest kinetic energy, and largest distance from lattice oxygen (3.8 eV), OH_br_ (2.6 eV) and OHt (1.7 eV) through the entire experimental course (Table 1). This may reflect the most stable presence of water molecules on the powder sample in various formats, including direct adsorption via the lone pair of water molecules, forming a complex network involving numerous hydrogen bonds among water molecules, OH_br_, OH_t_, etc. on different crystal planes^25^,^26^,^27^,^28^,^29^.

Of the two new peaks in phase 3, the first new peak was observed at 533.4 eV, and it coincides with the position of surface-adsorbed oxygen. Because oxygen was not supplied in this phase or observed on the sample during phases 1–2, the presence of surface-adsorbed oxygen could be due to water splitting through hole oxidation of water, generating hydrogen and oxygen (H_2_O + 2*hv* → H_2_ + ½O_2_)^30^; especially water splitting though photocatalysis over P25 has been reported^31^. The second new peak at 1.2 eV and 5.1 eV above the respective surface-adsorbed water and lattice oxygen is identified as the airborne water vapor supplied to the chamber (Table 1) (or BE 535.5 eV, Fig. 2), consistent with previously reported observations^5^. The airborne status of the water vapor was experimentally verified; when the XPS probe was pointed to the ambience of the study chamber, the species on the sample surface between 529.2–534.5 eV disappeared from the O 1s spectrum, whereas this peak remained (Supplementary Figure S2).

### Effects of both water vapor and O_2_ (Phase 4)

**Figure 3:**
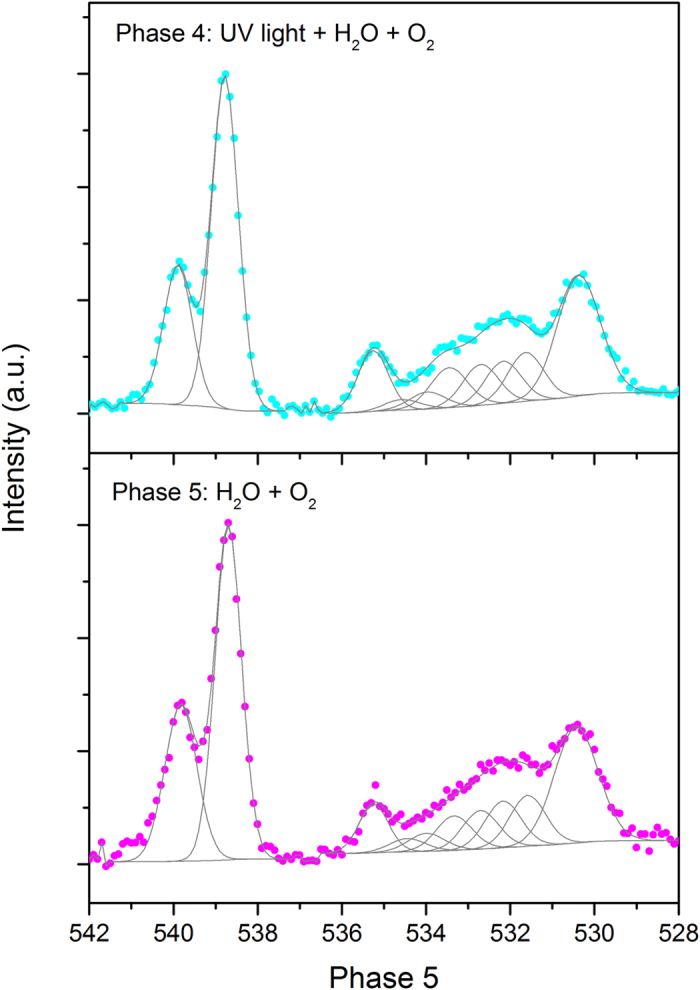
Enlarged spectra between BE 528.0–537.0 eV are provided for phases 4 and 5 to more clearly show the deconvoluted peaks.

The most drastic changes on the sample surface occurred under the supply of all components (UV irradiation, water vapor, and oxygen) in the study chamber during phase 4, a condition closer to diurnal environment. To better delineate the changes during phase 4, the deconvoluted peaks between 528–537 eV were enlarged as shown in Fig. 3 to counter the larger noise of the O 1s spectrum during phase 4 when the supply of both water vapor and O_2_ gas caused surface changes and scattering. Even with the lowered resolution, an overlay of the spectra of phases 3 and 4 verified significant surface changes in phase 4 (Supplementary Figure S3(a)). The addition of 0.8 Torr oxygen in this phase further lowered the peaks of Ti 2p_3/2_ by more than 0.1 eV. Although successive addition of water vapor and oxygen from phase to phase caused limited incremental change (more than 0.1 eV) relative to the initial conditions (phases 1 & 2), both core Ti 2p peaks decreased by approximately 0.2–0.3 eV, reaching the lowest BE in the entire experimental course (Fig. 1). This should be mainly attributed to the pressure factor on the Ti 2p peaks, which is later confirmed by the reversed position when all the gases supply was terminated in phase 6.

Along with the greatest decrease in Ti 2p peaks during phase 4, the O 1s spectrum is the most complex, comprising at least 10 species, including the three peaks consistently observed in the first three phases (lattice oxygen, OH_br_ and OH_t_), two new peaks at 532.2 and 534.6 eV emerging on the powder, surface-adsorbed oxygen (533.5 eV), surface-adsorbed water (Fig. 3), and three peaks at higher BE attributed to airborne components, that is, the supplied water vapor and oxygen (phase 4, Fig. 2). The newly supplied oxygen in this phase would result in signals of both the airborne species and O_2_ adsorbed on the powder surface, consistent with the position reported in the literature^32^. The gaseous oxygen is represented by two peaks (538.8 and 539.9 eV) that are ~1.1 eV apart (Fig. 2), reflecting the 1s level of spin splitting of the paramagnetic nature of O_2_^33^,^34^.

As observed via XPS for the first time, the presence of oxygen is required to form the two new surface peaks at 532.2 and 534.6 eV, which indicates them as respective peroxide species and hydrogen peroxide dissolved in surface water on the powder. According to the reaction mechanisms shown in Table 2 (R6–R10), when all components (UV, water and oxygen) were present (i.e., during phase 4), at least two new oxygenated species (HO_2_^**·**^ and H_2_O_2_) are expected. Chemically, these peroxides can be formed through the reactions of O_2_ + H^**·**^ (H^+^ + e_cb_^−^ → H^**·**^) → HO_2_^**·**^ and O_2_^**·**−^ + O_2_^**·**−^ + 2 H^+^ → H_2_O_2_ + O_2_ (R6 & R7, Table 2). Considering their high solubility in water, we propose that the peroxides on the powder could produce the peak at 532.2 eV, with their dissolved forms in water producing the peak at 534.6 eV (phase 4, Fig. 3). It is worth noting that although the published results indicate that the peak at 532.2 eV is more likely ascribed to surface-adsorbed H_2_O_2_ because of the transient status of HO_2_^**·**^ (or HO_2_) as a precursor of H_2_O_2_ and OH_t_^2^,^35^, more studies using other characterization tools (such as IR and STM) in the future are needed to identify the compounds involved, and hence this peak is assigned as a composite of peroxides in the discussion below.

With the supply of oxygen during phase 4, water molecules adsorbed on the sample surface decreased by 0.2 eV from their position in phase 3, drawing closer to the lattice oxygen, OH_br_ and OH_t_ with a difference of 3.6, 2.3, and 1.3 eV, respectively (Table 1). Concurrently, the position of water vapor also shifted closer to lattice oxygen and lowered its position by 0.2 eV, yielding a distance of ~1.3 eV above the surface-adsorbed water peak, which remained a constant under the changing environment through phases 3–5 (Table 1). This consistent movement under different study environments, reported for the first time, serves as a reference to identify surface-adsorbed water and is independent of its diverse behavior. This also indicates that the gas-surface interactions of water molecules may regulate or stabilize the overall study system. In other words, achieving such gas-surface interplay of water could be a primary criterion when the surface-adsorbed water molecules undergo complicated movements (e.g., dissociation and forming a complex hydrogen network), resulting in a net decrease of 0.2 eV.

The most pronounced decrease in the C 1s spectrum is noted during phase 4, with the peak area reduced by 70% relative to the initial phase 1, agreeing with the reported observation that upon exposure to oxygen, fewer organic compounds were detected on the sample surface^14^. The substantial disappearance of carbonaceous materials from the powder surface is expected because the major oxidants (e.g., O_2_, O_2_^**·**−^, OH^**·**^, HOO^**·**^, and H_2_O_2_) are most abundant during phase 4 with the presence of UV irradiation, water vapor, and oxygen, a condition relatively closer to the real environment.

### Termination of UV irradiation (Phase 5), water vapor and oxygen supply (Phases 6) followed by prolonged darkness (Phase 7) and the return of UV irradiation (Phase 8)

To present the new findings of various species in the last four phases, this section first discusses the significance of the Ti 2p spectra, which is supported by the O 1s species in phase 5. Then, the alteration in the surface-bound oxygen in phase 5 and the water adsorbed on the powder in phase 6 are discussed before other O 1s species are addressed. This section ends with the observation and implication of persistent peroxides on the powder through the latter stage of the experiment course.

The lack of change in the Ti core peaks during phase 5 indicates that turning off UV irradiation alone did not have a significant influence on the Ti 2p_3/2_. Rather, the continually supplied water and oxygen dominantly maintained the Ti 2p at the same position in the dark. During phase 6, an absence of pressure by terminating the supply of water vapor and oxygen in the dark reversed the decreasing trend in Ti 2p_3/2_ and increased the BE by 0.2 eV (phase 6, Table 1). This is the most substantial change in the Ti 2p spectra within a single phase throughout the entire experiment; it returned the spectrum to the original BE position of phase 1 (Fig. 1) by offsetting almost all the stepwise decrease in BE with the successive addition of UV irradiation, water vapor, and oxygen molecules during the initial four phases. The reverse trend in phase 6 also confirms the observation in phase 5 that the presence of water vapor and oxygen is the key factor in preserving Ti 2p peaks at the same position in the dark; hence, when the supply of water vapor and oxygen was terminated in phase 6, the pressure effect was also removed, returning the two Ti 2p peaks to their original BE. Maintaining the sample in a dark environment and under vacuum for 10.5 hours (phase 7), followed by re-irradiation of UV in phase 8, did not cause observable changes in the two core peaks, reflecting the catalytic (sustainable) nature of the TiO_2_ powder examined in this study.

The corresponding O 1s spectrum, which also remained similar to phase 4 (Supplementary Figure S3(b)), supports the unchanged position of the Ti 2p species in phase 5, demonstrating the persistent presence of O 1s species and affirming the lasting reactivity of TiO_2_ powder in the dark. This explains the observation of extended bacterial disinfection activity in the dark after the light irradiation used to activate the P25 powder was turned off^36^. Once activated, the water vapor and oxygen can maintain the reactivity of TiO_2_ powder without photons (for at least 30 min). This also suggests that photons are required to initiate, but unnecessarily always needed to sustain the reactivity of TiO_2_ powder. In addition to indicating the extended contribution of TiO_2_ powder to the nocturnal ambient environment, it could potentially save energy when photocatalytic TiO_2_ powder is used for various applications (e.g., photodynamic therapy) because light irradiation is not perpetually required.

Surface-adsorbed oxygen lowered its position by 0.2 eV in phase 5 (Table 1), suggesting weakened photon-involved processes such as photoadsorption, photodissociation, and photodesorption, and thus the decreased BE position. Nevertheless, oxygen persistently adsorbed on the sample surface, and its unwavering position at 2.9–3.0 eV higher than lattice oxygen under the changed environment of phases 5–8 (Table 1) is reported for the first time.

Relative to other species, the surface-adsorbed water on the powder (533.8 eV) exhibited the most notable change in the O 1s spectrum during phase 6, decreasing its position in phase 5 by 0.2 eV (Fig. 3). In fact, the surface-adsorbed water molecules dynamically responded to the changing environment in this work (Fig. 2). After reaching the highest BE position during phase 3, the addition of oxygen lowered the surface-adsorbed water by 0.2 eV, independent of the presence and absence of light during phases 4 and 5 (Fig. 2). An additional decrease by 0.2 eV occurred upon termination of the water and oxygen supply during phase 6, while surface-adsorbed water molecules remained at 3.3–3.4 eV above lattice oxygen throughout phases 6–8, consistent with the position during the initial two phases under a similar vacuum environment (Table 1). This phase also has the surface-adsorbed water closest to lattice oxygen, with the lowest BE position among all the studied environments. These dynamic changes reflect the net effects of complicated factors, such as the structure of the powder surface, water-surface interactions, surface species, and surface reactions^26^, affecting chemisorbed and physisorbed water molecules on the powder surface. While the reported position of the surface-adsorbed water molecules covered a wide range, 2.0–3.8 eV higher than lattice oxygen^5^,^14^,^15^,^16^, our results differentiate and specify the position of water adsorbed on the powder surface under individual environmental conditions.

The remaining O 1s species (lattice oxygen, OH_br_, OH_t_, H_2_O_2_ dissolved in water, and peroxides) on the powder surface reached their final position in phase 5 without further adjustment of the termination of the gas supply (phase 6), prolonged vacuum in the dark (phase 7) and resumed UV irradiation (phase 8) (Fig. 2). Although the pressure factor, which lowered the position of the three O 1s surface species (lattice oxygen, OH_br_ and OH_t_) during phase 3, should be reversed upon termination of the supply of both water and oxygen vapor in phase 6, the expected increase in the BE position could be offset by the reduction in organic compounds on the powder, uncovering some crystal planes for hydroxyl groups to re-arrange thereon. As discussed earlier, the oxidation during phase 4 was so strong that the subsequent C 1s spectra comprised few peaks; from phase 5 through the rest of the experiment, there are marginal differences in the surface organics, although some compounds could remain on the sample surface. Hence, it is not surprising that after the UV irradiation was resumed in phase 8, the effects of photolysis on the C 1s spectrum were not observable. The substantially reduced organic species on the sample surface during the later phases of this study were verified by overlaying the C 1s spectra of phases 1 and 7, which were obtained under a similar environment of vacuum in the dark (Supplementary Figure S1(c)). With fewer organic compounds on the powder, the position of OH_br_ is 1.1–1.2 eV above lattice oxygen, slightly lower than its position during phases 1 and 2 (Table 1). On the other hand, the position of OH_t_ relative to lattice oxygen was invariant to the reduction in surface organic contaminants and changes in the environment, remaining 2.1–2.3 eV higher than lattice oxygen throughout the entire experiment (Table 1). Taken together, differentiating OH_t_ from OH_br_ in all study phases provides a significant key to investigate photocatalytic reaction mechanisms of various compounds on the powder via XPS because the nature and composition of hydroxyl groups can alter the acidity, hygroscopicity and reactivity of the powder, affecting how various compounds interact with TiO_2_ surface in terms of adsorption, dissociation, oxidation, etc^37^.

The BE position of H_2_O_2_ dissolved in water remained at 4.0–4.2 eV and 2.3–2.4 eV above the respective lattice oxygen and surface peroxides since phase 5 (Table 1). This peak, however, disappeared from the system after prolonged vacuum treatment in the dark during phase 7. Unlike the disappeared H_2_O_2_ dissolved in water, peroxides persisted throughout the changing environment, with a consistent position of 1.7 eV above lattice oxygen since phase 5 (Table 1). It is worth noting that while the peroxide peak (532.2 eV) remained at the same position throughout the rest of the experimental phases (Table 1), its corresponding peak area significantly decreased by 60% after phase 6, suggesting its disappearance during prolonged exposure to vacuum in the dark during phase 7. Nevertheless, the persistence of this peak through phases 5–8 (Fig. 2) shows a unique characteristic of the extended oxidative capability of activated TiO_2_ in the dark. Unaffected by the terminated supply of both water vapor and oxygen, prolonged vacuum in the dark, and resumed UV irradiation, this peak remained on the surface, demonstrating the strong adherence (possibly chemisorbed) to the powder under varied environments. This can also explain the observed bio-inactivation exerted by TiO_2_ powder that was used without light activation^38^ because once activated, the oxidative capability is formed on TiO_2_ powder surface, almost like an inherent function.

## Conclusions

Changes in the Ti 2p, O 1s and C 1s spectra of the photocatalytic TiO_2_ powder surface under the systematic supply and removal of UV irradiation, water vapor, and oxygen in eight experimental phases enable direct evaluation of photocatalytic TiO_2_ powder surface under atmospheric relevant conditions vs. selectively controlled ones, and comparison under diurnal vs. nocturnal environments. Both the Ti 2p and O 1s spectra underwent hysteresis movement, reaching the lowest binding energy (BE) with the combined presence of UV irradiation, water vapor, and oxygen, a condition closer to real atmospheric environment. The decreasing trend in both spectra was not reversed by the removal of UV irradiation, but by the removal of the water and oxygen supplies in the dark, demonstrating that the presence of both water and oxygen is sufficient to sustain the catalytic capability of TiO_2_ powder without photons for a period of time. Water dynamics on the powder are characterized for individual study environments. When only water vapor and UV irradiation were supplied, the surface-adsorbed water molecules exhibited the highest BE, with the largest distance from the lattice oxygen, OH_br_ and OH_t_. Under a condition closer to diurnal and nocturnal atmospheric environments, the surface-adsorbed water molecules moved closer to these three O 1s species with a distance of 3.6, 2.3–2.4, and 1.3 eV, respectively, while having the shortest distance from them under vacuum (phases 1, 2, 6–8).

The following summarizes the characteristics of surface species that are consistent under all environments in this study, and will be applicable for diverse conditions, including artificially designed and atmospheric relevant environments:

1. OH_br_ and OH_t_ are identified at 1.1–1.3 and 2.1–2.3 eV above lattice oxygen;
2. When present, water vapor is always positioned 1.3 eV higher than the surface-adsorbed water molecules;
3. Peroxides, once formed, are positioned 1.7–1.8 eV above lattice oxygen;
4. Hydrogen peroxide dissolved in water, if present, is 4.0–4.2 eV and 2.3–2.4 −eV above respective lattice oxygen and peroxides on the powder surface; and
5. Surface-adsorbed oxygen is consistently positioned 2.9–3.1 eV above lattice oxygen.

Two new peaks at 1.7–1.8 eV and 4.0–4.2 eV above lattice oxygen were proposed as peroxides (532.2 eV) and hydrogen peroxides (H_2_O_2_) dissolved in water (534.4–534.6 eV) on the powder surface, respectively. The reactivity of TiO_2_ powder in the dark is experimentally evidenced by (1) the presence of water vapor and oxygen alone, conditions closer to a real nocturnal environment, retains the activated status of O 1s species, as well as the redox capability of activated TiO_2_ powder for a period of time, and (2) once formed, peroxides on the TiO_2_ powder persist through varied environments. These findings verify the proposed hypothesis, elucidate the reactivity of TiO_2_ powder in the dark reported in the published literature, and enable new applications of photocatalytic TiO_2_ powder.

## Methods

Commercially available TiO_2_ (Degussa P-25, EVONIK Industries AG, Germany) comprising 80% anatase and 20% rutile was employed to prepare powder samples for *in situ* XPS scans. TiO_2_ powder in ethanol underwent ultrasonic agitation to create a homogeneous colloidal solution. An aliquot of the solution was placed on a gold plate and dried. Prior to hosting TiO_2_ samples, the gold substrate was cleaned using an ethanol solution in an ultrasonic bath for 10 min. The prepared samples were placed in an analysis chamber for *in situ* XPS measurements at the 9.3.2 beamline of the Advanced Light Source at Lawrence Berkeley National Laboratory following the experimental set-up described in Grass *et al*.^39^. In brief, incident photon energy ranging from 250–850 eV was supplied by increasing the inelastic mean free path of the emitted photoelectron. This study primarily used incident photon energy of 650 eV for various XPS scans. The spectroscopy chamber was equipped with a differentially pumped electrostatic lens, allowing a maximal pressure of 1 Torr of various gases in the chamber at ~21 °C. A 9 LED blacklight flashlight (LEDwholesalers, USA) with a wavelength of 365 nm was positioned approximately 20 cm from the sample surface to activate the TiO_2_ samples.

**Figure 4:**
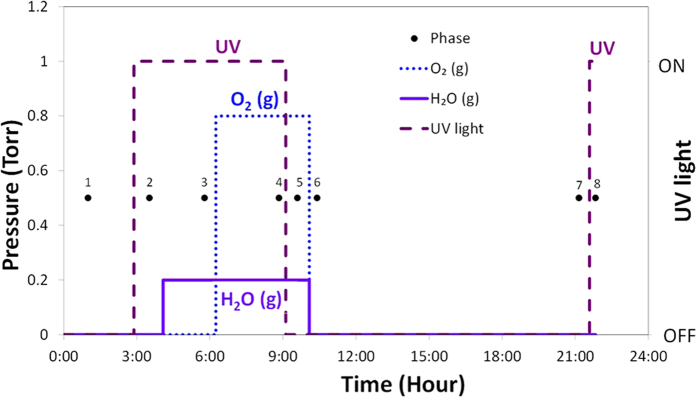
Schematic study environment for the *in situ* near ambient pressure XPS scans during individual eight study phases.

The TiO_2_ samples underwent *in situ* XPS scanning in eight consecutive phases under successively altered ambient conditions, as detailed in (Fig. 4), which were designed based on the commonly accepted photo-activation and chemical reaction mechanisms of TiO_2_ in the presence of UV irradiation, water vapor, and oxygen, as summarized in (Table 2). As control, the TiO_2_ sample was first scanned under vacuum in a dark environment (phase 1, (Fig. 4). This scan was conducted as a survey scan covering the range of 0–600 eV with a step of 0.2 eV; all other scans were performed at higher resolution of elements Au, Ti, C, and O, with a step of 0.1 eV. The scan during phase 2 was performed in the presence of UV light to activate TiO_2_ (phase 2, Fig. 4), forming electrons and holes (Table 2). The effects of water moisture on the activated TiO_2_ surface were captured during phase 3 (Fig. 4), where 200 mTorr of water (approximately 1% RH at 22 °C, or 0.0108 mole/m^3^) was pumped into the chamber for 75 min. This was followed by the addition of 800 mTorr of oxygen (approximately 0.0434 mole/m^3^) during phase 4 (Fig. 4), making up a total of 1 Torr of gas in the study chamber. Soon after the UV light was turned off, the TiO_2_ powder on the Au substrate was scanned to examine the immediate effects (phase 5, Fig. 4). After both the water vapor and oxygen supply were terminated in the dark for ~50 min, a scan was conducted to evaluate the changes in the TiO_2_ powder under vacuum and in a dark environment (phase 6, Fig. 4). Phase 7 allowed the powder sample to rest under a dark, vacuum environment for 10.5 hours (phase 7, Fig. 4) to observe the changes in various oxidants generated from the previously activated TiO_2_. The entire experimental course was completed by resuming the UV light irradiation in the system (phase 8, Fig. 4) to compare with the results of scan 2, showing activated TiO_2_ prior to successive changes in the chamber ambience.

The Ti 2p, O 1s and C 1s spectra were processed with CasaXPS software (Casa Software Ltd., UK) employing a Shirley background correction. The sample spectra were corrected for charging effects using the Au 4f_7/2_ peak at 83.8 eV, which was consistent and stable through all changes in the scanning chamber. Individual peak areas were also normalized by the corresponding reference (Au) peak area during the same phase to account for the inherent influence during individual study phases. Consistent with the previously reported system error (0.1–0.2 eV) of the *in situ* near ambient pressure XPS system, an error of 0.15 eV in this study was determined based on the maximum variation in the difference of the binding energy between the lattice oxygen and the Ti 2p peak from one experimental phase to another. Peak deconvolution was performed using the CasaXPS software, fitting multiple Gaussian-Lorentzian components in the ratio of 85:15. The full width at half maximum (FWHM) for all peaks obtained under an incident photon energy of 650 eV was constrained with a variation of 0.1 eV. The procedure and rationale of peak fitting for the spectra in detail is available in the supplementary materials.

## Additional Information

**How to cite this article:** Krishnan, P. *et al*. Characterization of photocatalytic TiO**2** powder under varied environments using near ambient pressure X-ray photoelectron spectroscopy. *Sci. Rep.* **7**, 43298; doi: 10.1038/srep43298 (2017).

**Publisher's note:** Springer Nature remains neutral with regard to jurisdictional claims in published maps and institutional affiliations.

## Supplementary Material

Supplementary Information

## Figures and Tables

**Table 1:** Discussed binding energy (eV) and relative position of the Ti 2p and O 1s species during individual study phases.

Species	Study phase							
	1	2	3	4	5	6	7	8
Surface adsorbed species								
Ti 2p_3/2_	459.3	459.3	459.1	459.0	459.0	459.2	459.2	459.2
Lattice O^a^	530.7	530.6	530.4	530.4	530.4	530.5	530.5	530.5
OH_br_	1.2	1.2	1.2	1.3	1.2	1.2	1.1	1.1
peroxides				1.8	1.7	1.7	1.7	1.7
OH_t_	2.2	2.1	2.2	2.3	2.3	2.2	2.2	2.3
O_2_			3.0	3.1	2.9	2.9	2.9	3.0
H_2_O	3.3	3.4	3.8	3.6	3.6	3.4	3.3	3.3
OH_br_^b^	2.2	2.2	2.6	2.3	2.4	2.2	2.2	2.2
OH_t_^c^	1.2	1.2	1.7	1.3	1.3	1.1	1.2	1.0
H_2_O_2(l)_				4.2	4.0	4.1		
Airborne species								
H_2_O_(g)_			5.1	4.9	4.8			
O_2(g)_				8.4	8.3			
O_2(g)_				9.5	9.4			

^a^The reference for the relative position of other O 1s species shown below.

^b^The BE distance between surface adsorbed water molecules and OH_br_.

^c^The BE distance between surface adsorbed water molecules and OH_t_.

**Table 2:** Photocatalytic activation mechanism of TiO_2_.

Reaction		Reference
R1	TiO_2_ + *hν* → e_cb_^−^ + *h*_vb_^+^	40
R2	e_cb_^−^ + *h*_vb_^+^ → heat	41
R3	*h*_vb_^+^ + H_2_O → ^**·**^OH + H^+^	42
R4	*h*_vb_^+^ + OH^−^ → ^**·**^OH	43
R5	e_cb_^−^ + O_2_ → O_2_^**·**−^	41
R6	O_2_^**·**−^ + O_2_^**·**−^ + 2 H^+^ → H_2_O_2_ + O_2_	41
R7	O_2_ + H^**·**^(H^+^ + e_cb_^−^ → H^**·**^) → HO_2_^**·**^ O_2_^**·**−^ + H^+^ → HO_2_^**·**^	41, 44
R8	HO_2_^**·**^ + H^+^ + e_cb_^−^ (H + HO_2_) → H_2_O_2_	45
R9	H_2_O_2_ + *hν* → 2^**·**^OH	46
R10	H_2_O_2_ + e_cb_^−^ → ^**·**^OH + OH^−^	41

*hν*: photons, *h*_vb_^+^: valence band hole, e_cb_^−^: conduction band electron, ^·^OH: hydroxyl radical, OH^−^: hydroxide, O_2_^·−^: superoxide radical, HO_2_^·^: hydroperoxyl radical, H_2_O_2_: hydrogen peroxide.
