# Supplementary material for: Characterization of photocatalytic TiO2 powder under varied environments using near ambient pressure X-ray photoelectron spectroscopy
Source: Sci Rep. 2017 Feb 27;7:43298. doi: 10.1038/srep43298 (PMC5327435; doi:10.1038/srep43298)
Supplement: Supplementary Information [file srep43298-s1.pdf]

Supplementary Information

Characterization of photocatalytic TiO_2 powder under varied environments using near ambient pressure X-ray photoelectron spectroscopy

Padmaja Krishnan[1], Minghui Liu[1], Pierre A. Itty[2], Zhi Liu[3,†], Vanessa Rheinheimer[1], Min-Hong Zhang[1], Paulo J. M. Monteiro[2] & Liya E. Yu[1,*]

[1]Department of Civil and Environmental Engineering, National University of Singapore, Singapore 119260, Singapore
[2]Department of Civil and Environmental Engineering, University of California, Berkeley, California 94720, USA
[3]Advanced Light Source, Lawrence Berkeley National Laboratory, Berkeley, California 94720, USA

*Corresponding author. Tel: +65 6516 6474; *E-mail address:* liya.yu@nus.edu.sg
† Present address: Shanghai Institute of Microsystem and Information Technology, Chinese Academy of Sciences, China

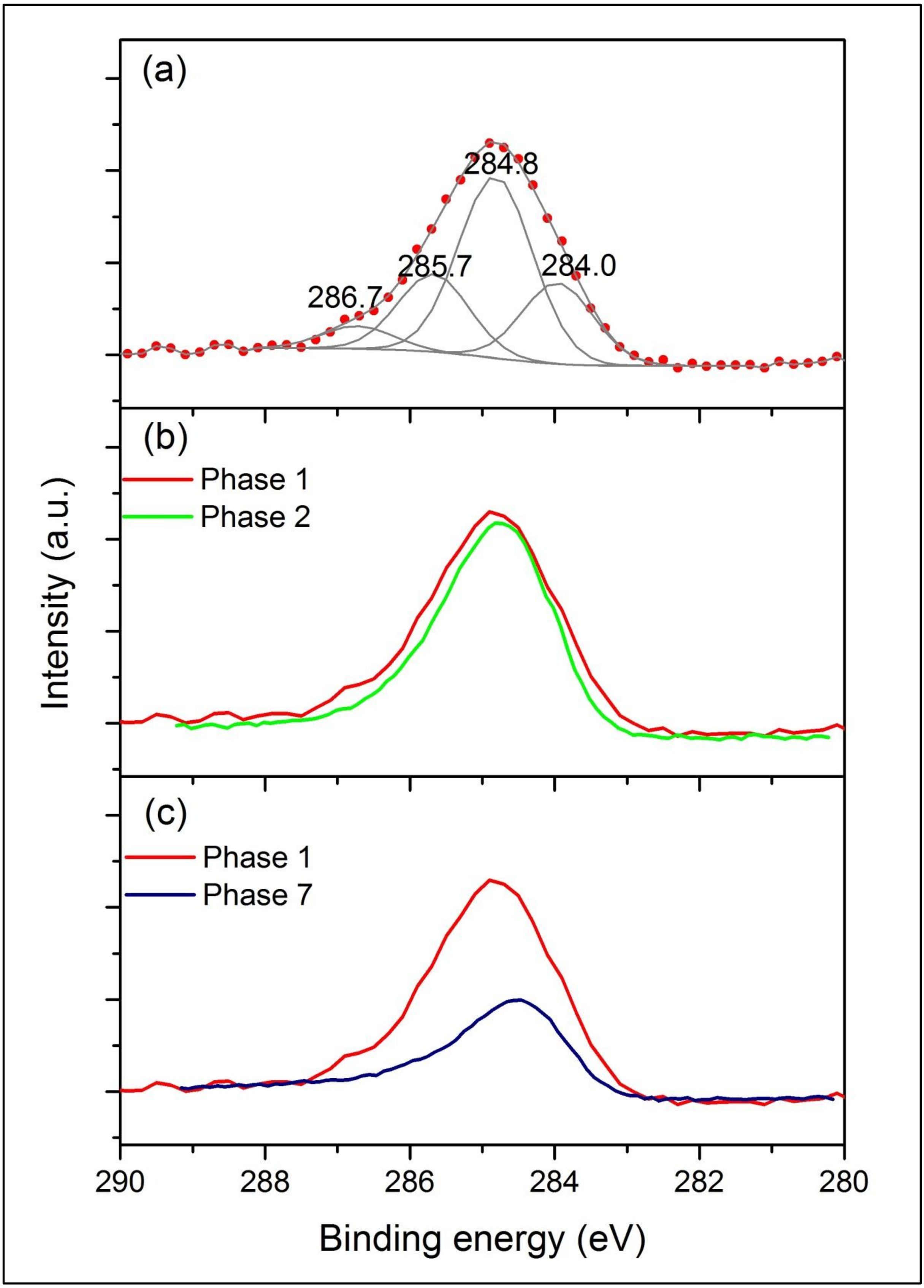

Figure S1. Processed C 1s spectra for (a) deconvoluted peaks during phase 1, and for comparison of C 1s spectra of (b) study phases 1 and 2, and (c) study phases 1 and 7.

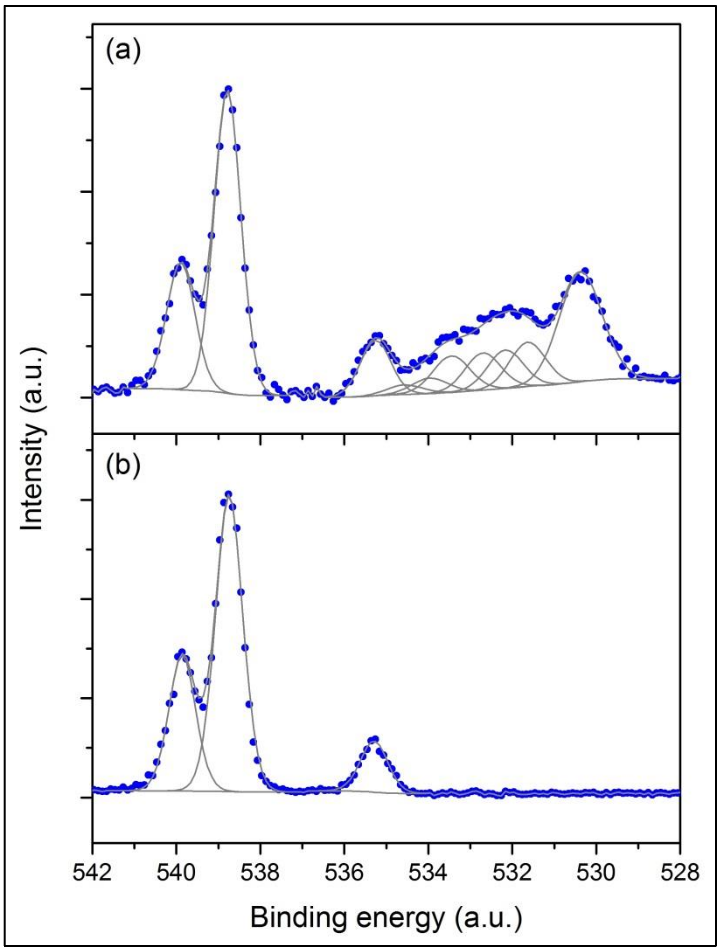

Figure S2. O 1s XPS spectra of (a) sample surface, and (b) study ambience.

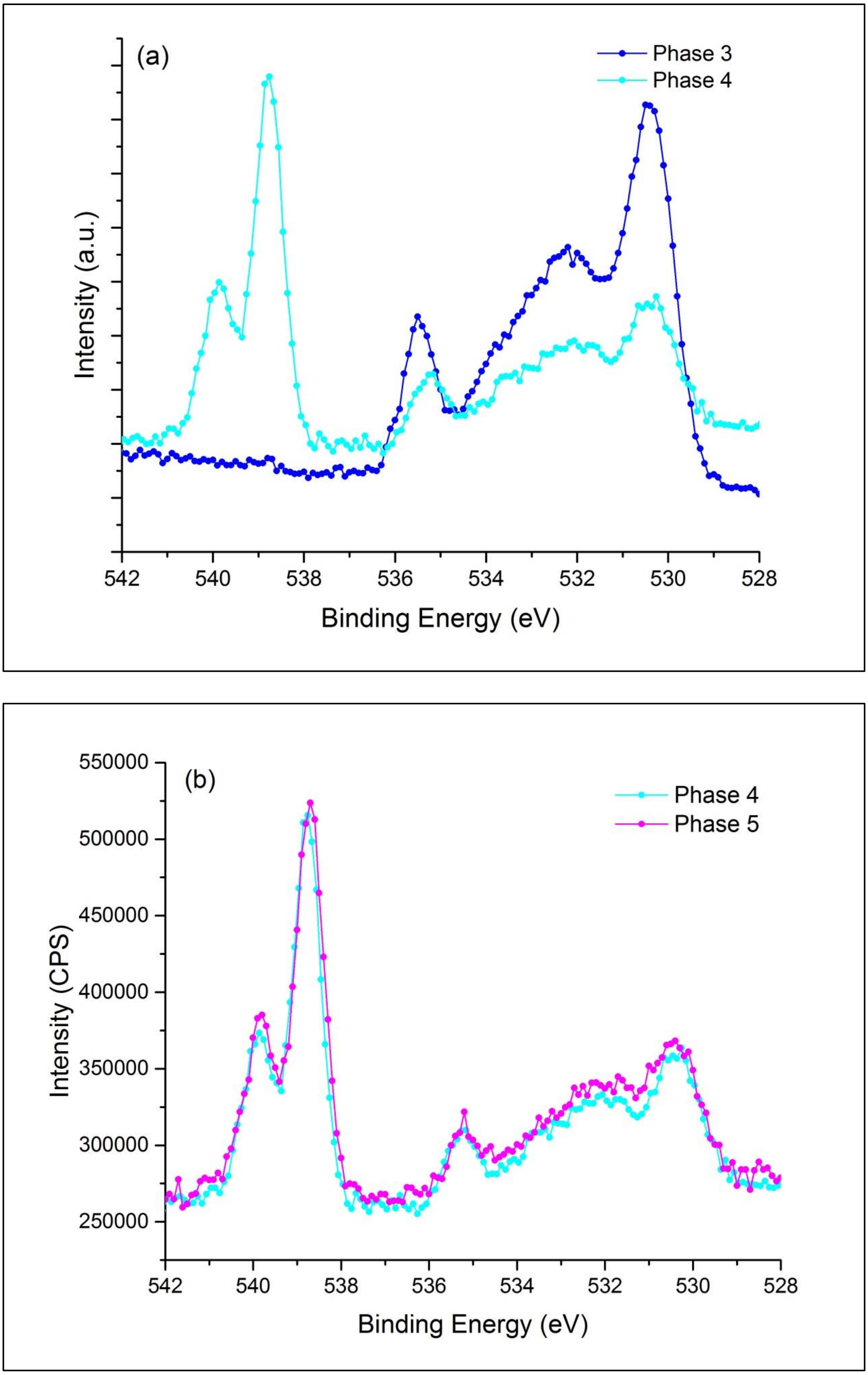

Figure S3. Overlay of the O 1s XPS spectrum of (a) phase 3 and phase 4, and (b) phase 4 and phase 5.

1. Peak deconvolution

The spectrum in individual phases was deconvoluted by following a consistent protocol and evaluation. We first identified and fixed the position of certain compounds based on comparable study conditions and consistent BE positions reported in the published literature (e.g. 530.6 eV for lattice oxygen under vacuum and in the dark). Then, peak fitting was performed for other species with the corresponding residual errors recorded. The same peak fitting exercise for each individual study phases was repeated for multiple times to generate various scenarios. The finalized peak fitting was selected when the consistent FWHM of the major peaks among 8 study phases can be obtained with a minimal residual error, and supported with meaningful rationales (i.e. priori knowledge obtained from published literature).

As an example, Table S1 below shows the data demonstrating how the peak fitting was evaluated and determined for phases 4 and 5. In brief, a 6-peak fitting broadened the FWHM of OH_{br} by 50% (from 1.0 to 1.5) during phase 4 and significantly shifted the peak of surface adsorbed water molecules during phase 5 (Table S1) without supporting scientific rationales. The former suggests that more peaks should be segregated for the spectrum of phase 4, and the latter indicates that other peak-fitting for the phase-5 spectrum should be more suitable. When the 8-peak fitting was performed, the FWHM of the OH_{br} during phase 4 becomes satisfactory and the surface adsorbed water molecules during phase 5 can be allocated at a reasonable position (Table S1), demonstrating the necessary presence of two more species during phases 4 and 5. In addition, the established chemical reactions of photocatalytic reactions of TiO_2 (Table 1) and literature results[2,35] show that the presence of both surface peroxides and $H_2O_{2(l)}$ should be expected when water vapor and O_2 are supplied. Hence, it is meaningful of assigning these additional two peaks to peroxides and $H_2O_{2(l)}$ on surface during phases 4 and 5. Nevertheless, as specified in the relevant discussion in the

main text, although the published literature studies support the assignments of the two additional peaks, more studies using other characterization tools (such as IR and STM) in the future are needed to identify the compounds involved.

Table S1. Comparison of 6 vs. 8 peak for phases 4 and 5.

	Phase 4				Phase 5			
	8 peaks		6 peaks		8 peaks		6 peaks	
	BE	FWHM	BE	FWHM	BE	FWHM	BE	FWHM
**Lattice O**	530.4	1.2	530.3	1.2	530.4	1.2	530.3	1.2
**OH_{br}**	531.6	1.0	531.7	1.5	531.6	1.0	531.6	1.6
**Peroxides**	532.2	1.0			532.2	1.0		
**OH_t**	532.7	1.0	532.5	1.4	532.7	1.0	532.5	1.3
**O_2**	533.5	1.0	533.5	1.1	533.3	1.0	533.5	0.9
**H_2O**	534.0	1.0	534.1	1.0	534.0	1.0	534.3	1.0
**$H_2O_{2(l)}$**	534.6	1.0			534.4	1.0		
**$H_2O_{(g)}$**	535.3	0.9	535.2	0.9	535.2	0.8	535.2	0.8
**$O_{2(g)}$**	538.8	0.8	538.8	0.8	538.7	0.7	538.7	0.7
**$O_{2(g)}$**	539.9	0.8	539.9	0.8	539.8	0.9	539.8	0.9
